# Is Morality a Product of Law?

**Published:** 1898-02

**Authors:** 


### Editorial.

### IS MORALITY A PRODUCT OF LAW?

This query came forcibly to the mind of the writer when the editorial in the *Dental Cosmos* for January was read. It assumes to be an answer to an "open letter" from the pen of Dr. B. F. Arrington in relation to examining boards, and, in addition, our esteemed contemporary paid special attention to an editorial in the same number of that journal.

It is unnecessary for the writer to take up the defence of the subject-matter in the open letter, for the writer thereof is fully capable of defending his own positions if deemed necessary. The question seems to have a broader outlook than a mere controversy in regard to boards of examiners, and resolves itself, naturally, into the more serious phase of law in its relations to morals, and directly as a powerful means for advancing civilization, if what is claimed for it be true.

It is trite to say that law is an outgrowth of an effort of society to protect itself. The older the civilization the more thoroughly has social life entrenched itself with statutes. This is so true of the countries of Europe that, whether the government be monarchical or republican, custom demands the same regulations, the same everlasting surveillance of the police, the same complexity in legal forms until the freedom of the individual, whether under king or president, is practically annihilated.

From the dawn of civilization the human race has been gradually forging the chains of its own enslavement. This, while true, does not by any means lead to the anarchistic idea that all law is wrong. A world without law would be an unthinkable problem. And a civilization without statutes would be anachronism, for it would cease to be civilization and at once become the perfected type of barbarism.

This being granted, the question naturally presents itself, If law has thus from the earliest times been used as a promoter of good, has it not at the same time been a power in developing the moral side of humanity? The answer might be that indirectly it has been the means of increasing the comforts of life, supporting the weak and curbing the strong, and punishing the evil-doer. While it is possible that this would be the answer of the majority, it is by no means certain that enactments are to be credited with this improvement in the condition of all civilized peoples. Statutes are, or should be, the outgrowth of a popular need, but, unfortunately, they are more often the demand of the despotic will of a single individual or of the equally despotic power of a minority under a republican form of government. It is then proper to conclude that law within proper limits is a necessity, but that it never did and, it is safe to assume, never will make mankind moral through its stringent enactments. Until mankind comprehends the whole law of development and has studied evolution through all its changes, for good or ill, from the savage to the highly developed and perfectly rounded-out human creation, will degeneracy be a factor in human life and for which no remedy will be found in legal enactments.

The prisons, insane asylums, idiot asylums, and the entire list of constantly multiplying evidences, are an ever-present reminder that in respect to law civilization is a stupendous failure. It has been from time immemorial an attempt to remove a disease by force when the cause finds its root in the very origin of the race.

If these views be admitted, then it seems to the writer that this almost insane demand for law and more law should be brought to the test of reasonable discussion, for if there is any one thing to-day that needs the light it is the demand, constantly made, for an increase of law to effect a higher moral standard in the dental profession. If it has not this meaning, then it can have no value.

It is apparently the effort to bring all to a certain standard of ability, and thus to drive to oblivion all empirical workers fattening on the gullibility of the general public.

If this were possible, then law would gratefully be recognized as the most valuable adjunct of professional excellence; but, alas, the promoters of statutes forget the fact, briefly alluded to, that laws, severe or otherwise, are powerless to increase the moral force of the individual, and that therefore, in spite of these and their drastic conditions, the empirical exhibitor of show-cases will establish his institute of cheap wares in all the marts of civilization with more effrontery than ever.

It is a recognized fact with all conversant with college work that in every class there will be found several degenerates, who, while morally deficient, may possibly be brilliant intellectually. There is no power in faculties to determine this, and these men can pass out into the world easily escaping the net laid for them in the examining boards, and at once become the terror of respectable practitioners and the mortification of the college from which they have received their diplomas. The powers of evil are apparently wiser in their generation than the children of light, for they have taken the dental profession at its own standard, and are preparing themselves to meet all laws and all boards of examiners, in order that they may carry forward the detestable work for which they are morally fitted. This has become a notorious fact, and no law, no college standard, and no re-examinations will suppress these marauders upon the character of a reputable calling. It is said, and we presume with some degree of truth, that notwithstanding the State of New York has one of the most stringent laws, carried out to the letter by those deputed to enforce its provisions, that more empirical practitioners in dentistry are to be found in the metropolis than at any former period. This should be the legitimate result.

The older civilizations have practically given up the contest. Especially is this so in Germany, where freedom of trade was legally established many years ago, including dental practice, without the title. Hence the great growth of mechanical dentistry with its societies and literature casting in the shade, in quantity at least, the real professional work of the Zahnarzt.

The first appearance of law upon our statute-books regulating dentistry was born in Alabama in 1841. New York was the first State after Alabama to procure a special legislative enactment regulating the practice of dentistry in 1868. One month later Ohio passed a State law, and in August, 1872, Georgia passed a similar law, followed by New Jersey in 1873, Pennsylvania in 1876. These enactments counted for little and made but slight impression upon the dental mind prior to 1875. The rush to acquire dental knowledge had not then begun; in fact, the dentists of the period were by no means equal in numbers to the rapid increase of population. The craze for law was intensified about the period of 1874, and after great difficulties some crude statutes were fastened upon our law-books, and then began the most remarkable demand, on the part of young men, for dental training. It is not probable that law had all to do with this, but it was coincident with the adoption of these stringent measures. From that day to this the rush for college instruction has not ceased, and from present appearances is likely to continue.

The editor of the *Dental Cosmos*, viewing this state of affairs, attributes it all to the growth of intelligent law, and very gravely asserts that "the salvation of our educational system depends upon the enactment of such legislative safeguards as will conserve our highest ideals and *prevent the licensing of practitioners who are a disgrace to their calling.*" (Italics ours.) He in another place says, "The proposition to do away with examining boards is a proposition to do away with legal restraint,—to do away with legal restraint is to establish anarchy." It is possible that years are not sufficient to carry the experience of the editor back to the period when every dentist was a law unto himself; when quackery had nothing to fear from officers of the law; when colleges knew nothing of a National Association of Dental Examiners; when graduates had only to dread the one examination. Then it was that anarchy reigned supreme, if the editor of the *Dental Cosmos* is to be credited, and every man's hand was raised against every other man. If it be true that the abrogation of all legal restraint would result as depicted, then it must have been the case when the profession was free from all law.

Fortunately for dentistry the pessimistic picture of the editor of the *Dental Cosmos* had no existence when freedom of professional action existed. The men who practised dentistry then were as truly infused with professional spirit as now, indeed more so. The practitioners of that day were as skilful as those of the present. The lines of professional ethics were as closely drawn then as now. It is folly then to assume that dentistry to-day is dependent on law for its very existence.

Our contention is not against law in general or the law of any

State in particular. The protest made here and in previous editorials is against the extension of a simple law regulating practice such as the original law of Pennsylvania. It is idle to assert that the present law, so much extolled, gives the power to find out the competency of the examining board. It practically does no such thing. It does give the right to examine the questions, and the council cannot go behind the report of the board, but "shall issue forthwith to each applicant who shall have been returned as having successfully passed said examination, a license to practise dentistry in the State of Pennsylvania." No power is given the council to decide upon the merits of the examination, whether it be good or bad. It does give the right to all interested to examine the questions, and as far as this goes it is an excellent provision, but suppose the questions are not what they should be. What then? Is there any power existing anywhere to displace these officials? Fortunately for Pennsylvania it has, at present, an excellent board, but it does not follow that this will be continued in the future.

The editor of the *Dental Cosmos* is on record for a decided opposition to the crude attempts of the National Association of Dental Examiners, and in his very drastic editorial, with the title of "Our National Beggar on Horseback," he demonstrated that he then possessed a keener appreciation of dabblers in law than he possessed when he penned his last editorial. Times change and men change with them, but, in the opinion of the writer, the law of right and justice endureth forever. We can make our standards for entrance to colleges as high as may be deemed advisable, but let not the graduating period of the hardly worked student, after his years of service, be made miserable by a body of men who neither know of what he may have endured or care for the result, whether that may be disastrous or not. It is time, as has before been asserted, that colleges were up and doing to protect their students from these law-makers who profess to believe that good men are made by legal enactments and that saints are the peculiar product of legislatures.

